# Oral candidiasis in liver transplant patients: species identification and antifungal susceptibility profile

**DOI:** 10.31744/einstein_journal/2024AO0138

**Published:** 2024-05-06

**Authors:** Clarice Elvira Saggin Sabadin, Daniel Archimedes Da Matta, Lísia Hoppe, Fernanda Aparecida Vieira Fernandes, Analy Salles de Azevedo Melo, Lilian Rigo, Dulce Aparecida Barbosa

**Affiliations:** 1 Universidade Federal de São Paulo São Paulo SP Brazil Universidade Federal de São Paulo, São Paulo, SP, Brazil.; 2 Meridional College Passo Fundo RS Brazil Meridional College, Passo Fundo, RS, Brazil.

**Keywords:** Antifungal agents, Candida, Candidiasis, Liver transplantation, Candidiasis, oral

## Abstract

**Objective:**

This study aimed to verify oral candidiasis, identify the causative species, and investigate the antifungal susceptibility of yeasts isolated from liver transplant patients.

**Methods:**

A descriptive analysis of 97 patients who underwent liver transplantation was conducted at a hospital. Two clinical examinations (Collections A and B) of the oral cavity were performed. Oral material was collected from all patients, inoculated in Sabouraud Dextrose Agar, and incubated at 35℃ for 48 hours. Samples were identified by molecular sequencing of the internal trascribed space region of rDNA.

**Results:**

An antifungal susceptibility test with fluconazole, amphotericin B, and micafungin was performed using the Clinical and Laboratory Standards Institute yeast broth microdilution method. Among the patients, 15 presented with oral candidiasis: eight in Collection A and seven in Collection B. The primary type of candidiasis was atrophic, followed by pseudomembranous candidiasis. The most prevalent species was *Candida albicans* (nine), followed by *Candida glabrata* (three), *Candida tropicalis* (two), and *Candida dubliniensis* (one). Regarding susceptibility to fluconazole, of the 15 samples, 11 were susceptible, three were susceptible in a dose-dependent manner, and one was resistant.

**Conclusion:**

The most commonly identified type of candidiasis was atrophic, with *C. albicans* and *C. glabrata* being the most prevalent causative species. One fluconazole-resistant isolate each of *C. tropicalis* and *C. albicans* were identified.

## In Brief

Sabadin et al. demonstrated that most yeasts isolated from patients who have undergone liver transplantation are susceptible to the antifungal agents fluconazole, amphotericin B, and micafungin. Fifteen patients presented with either atrophic or pseudomembranous oral candidiasis. *Candida albicans* and *Candida glabrata* were the most prevalent in these patients

## Highlights

Fifteen liver transplant patients presented with oral candidiasis.

The two prevalent types of candidiasis were atrophic and pseudomembranous

*Candida albicans* and *Candida glabrata* were the most prevalent species

Most isolates were susceptible to antifungal agents.

## Graphical abstract

**Figure:**
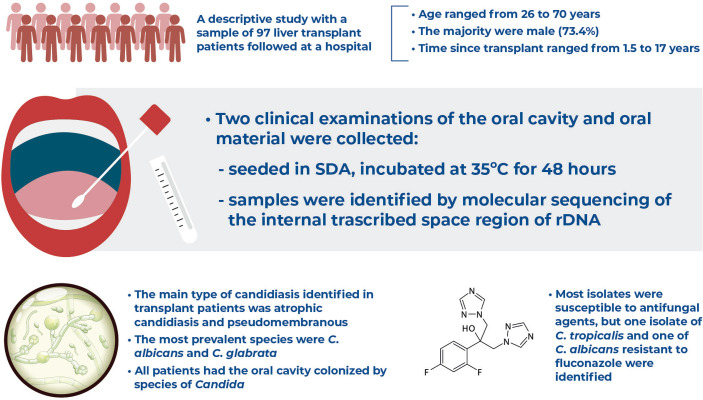

## INTRODUCTION

Organ transplantation remains one of the most significant advances in modern medicine. In many cases, it is the only therapeutic option for treating diseases related to end-stage organ failure.^(1)^ In recent decades, the occurrence of superficial and invasive fungal infections related to deaths and postoperative failure in transplant recipients has increased significantly, with candidiasis being the most common.^(2)^ Organ recipients must use immunosuppressive therapy to prevent rejection; however, these drugs can harm the oral health of some patients, creating conditions conducive to infections.^(1,3)^ Oral candidiasis is frequent in liver transplant recipients since these patients represent a higher risk category for oral infections due to the use of drugs that tend to weaken the immune system, as well as to the delay in diagnosis owing to the non-specificity of symptoms and methods for current diagnosis.^(4)^

*Candida* species are generally present as commensals in the oropharyngeal and gastrointestinal tracts and can cause a broad spectrum of serious diseases, ranging from local infections to disseminated diseases.^(5,6)^They are responsible for severe infections with a high rate of morbidity and mortality.^(7-9)^ Lesions in the oral cavity may present with different characteristics, such as pseudomembranous, atrophic, hyperplastic, and angular cheilitis, which may be accompanied by pain and a burning mouth sensation.^(10)^

*Candida albicans* is the most common species that causes oral candidiasis. However, infections by other *Candida* species have been reported to be increasing in frequency. The most important include *C. parapsilosis, C. glabrata, C. krusei*, *C. lusitaniae, C. tropicalis, C. dubliniensis*, and *C. auris.*^(9,11)^ Several of these species are resistant to commonly used antifungal drugs, and some have been proven to be highly virulent. Resistance to antifungal therapy in some *Candida* species makes managing oral candidiasis difficult.^(4,12)^

Knowledge and epidemiological data regarding the identification of *Candida* species and susceptibility to antifungal agents in patients who have undergone a liver transplant remains limited. Accurate identification of agents, antifungal resistance testing, and epidemiological surveys are essential for guiding therapy and determining the prognosis of fungal diseases.^(13,14)^ This research is of extreme clinical importance because invasive fungi have emerged as a leading cause of disease in patients with compromised immune systems. Lack of knowledge of the species and sensitivity to antifungal agents may lead to delays in proper intervention.

## OBJECTIVE

This study aimed to verify the presence of oral candidiasis, identify the species involved, and investigate the antifungal susceptibility profiles of yeasts isolated from patients who have undergone liver transplantation.

## METHODS

### Study participants and sampling

All the participants involved provided written informed consent. Ninety-seven patients who underwent liver transplantation and attended the transplant outpatient clinic of the *Hospital São Vicente de Paula,* Passo Fundo, RS, Brazil, between May 2017 and May 2019 participated in the study. The inclusion criteria were age >18 years, six months post-transplantation, and no use of antifungal agents. Sociodemographic data were recorded, and clinical examinations were performed on all patients who met the inclusion criteria for verifying infection in the oral cavity.

Oral material was collected twice after transplantation in all patients, within six months between them, with the first collection referred to as Collection “A” and the second as Collection “B”. Patients who did not present with oral candidiasis in any of the collections were excluded from the study, the results of which have been previously published by this working group.^(15)^

This study was approved by the Ethics and Research Committee of *Universidade Federal de São Paulo* (CAAE: 50413815.2.0000.5505; # 1.319.387).

### Yeast isolation and molecular identification

Oral material from all patients was collected using a sterile swab and inoculated on Sabouraud Dextrose Agar (SDA) (Becton Dickinson & Co. Sparks, MD, USA) with chloramphenicol and incubated at 37 ℃ for 48 h. Samples that produced yeast colonies were stored at -80℃ in a YPD medium (1% yeast extract (BD), 2% BCA to peptone (BD), and 2% glucose-containing glycerol (15% p/v)).

Yeast cells stored at -80°C were inoculated on CHROMagar^TM^ *Candida* plates (CHROMagar Microbiology, Paris, France) for 48 h at 37℃ to obtain pure colonies and presumptive identification of *Candida* spp. Molecular identification of *Candida* species was performed by sequencing the ITS region of rDNA. The genomic DNA of the isolates was extracted using the Ultra PrepMan Reagent^®^ (Applied Biosystems, Inc., Foster City, CA, EUA) according to the manufacturer’s instructions. Polymerase chain reaction (PCR) for amplification of the ITS region was performed using primers V9G (5’-TTACGTCCCTGCCCTTGTA-3’) and LS266 (5’-GCATTCCCAAACAACTCGACTC-3’).^(16)^ In the sequencing reaction, the same primers were used. Sequencher software (version 4.1.4) used two to four reads to obtain the consensus sequence. The sequences obtained were cross-referenced with the NCBI GenBank (https://www.ncbi.nlm.nih.gov/) using the nucleotide Basic Local Alignment Search Tool (BLASTn) to identify the isolates. An e-value <10^5^ and identity ≥98% were considered accurate parameters for species identification.

Green colonies grown on CHROMagar^TM^ *Candida* were subjected to molecular identification to differentiate between *C. albicans* and *C. dubliniensis*. The amplification of genomic DNA with species-specific primers for each species was performed as described by Ahmad et al. with slight modifications. For the amplification of the DNA fragments, 2µL of CALF primers (5›-TGGTAAGGCGGGATCG-CTT-3’) + CALR (5’GGTCAAAGTTTGAAGA TATAC) for *C. albicans* and CDUF (5’AAACTTGTCACGAGATTATTTTT) + CDUR (5’AAAGTTTGAAGAATAAAAA) TGG GC-3’) for *C. dubliniensis* were used at a concentration of 20µmol; 2µL of template DNA and 100µmol of each dNTP. Primer pairs were used to identify *C. albicans* and *C. dubliniensis* for separate PCR reactions. C-reactive protein products were analyzed by 2% agarose gel electrophoresis. The results were interpreted visually to verify the presence and size of the amplified fragments; 100- and 325-bp fragments were used to identify *C. albicans* and *C. dubliniensis*, respectively.^(17)^

### Antifungal susceptibility test

Antifungal susceptibility tests were performed according to the Clinical and Laboratory Standards Institute (CLSI) protocol M27ED4^(18)^ using the broth microdilution technique against the antifungal agents fluconazole, amphotericin B (SIGMA Pharma, USA), and micafungin (Astellas Pharma). Antifungal dilutions were performed with RPMI 1640 medium to obtain final concentrations ranging from 0.03-16μg/mL for amphotericin B and micafungin and from 0.125-64μg/mL for fluconazole.

The reading criteria included the minimum inhibitory concentration (MIC), which is the lowest concentration capable of visually inhibiting 50% of the growth of *Candida* spp. after treatment with fluconazole and micafungin and 100% after treatment with amphotericin B. Susceptibility test readings were performed 24 hours after incubation and interpreted following the CLSI document M6028^(19)^ to determine the clinical breakpoints. According to this document, there is no defined breakpoint for amphotericin B. Therefore, the clinical breakpoint used in this study was described by Nguyen et al.^(20)^

### Statistical analysis

The data obtained were analyzed using IBM SPSS™ (version 20.0, Armonk, New York, USA).

## RESULTS

Of the 97 patients included in this study, 15 (15.5%) presented with oral candidiasis. The results of subsequent analyses were obtained from these 15 patients. The age range of the patients was 26 to 70 years, with an average of 57.2 (DP ±10.5). Most patients were male (73.4%), and post-transplantation time ranged from 1.5 to 17 years, with an average of 8.6) years. Hepatitis C was the main reason for transplantation (40%), followed by hepatitis B (33.5%). Sixty percent of the patients had skin lesions at the time of collection, 46.7% had infectious diseases in the post-transplantation period, and six had bacterial infections. All patients used cyclosporine as an immunosuppressant, and 80% used partial or total dentures.

Sample collections took place in two stages, with an interval of six months between them. In Collection A, eight of 15 patients presented with candidiasis, five had atrophic candidiasis (CA), and three had pseudomembranous candidiasis (PC). In Collection B, seven patients presented with candidiasis; five had AC, and one had PC. Patients not infected in one of the collections were colonized with yeast in the other collection.

The yeasts were identified using molecular sequencing. Of the five AC cases from Collection A, three were caused by *C. glabrata*, one by *C. albicans*, and one by *C*. *tropicalis*. Of the three PC cases, two were caused by *C. albicans* and one by *C. tropicalis*. Four of the five AC cases in Collection B were caused by *C. albicans*, one by *C. dubliniensis,* and 100% of the PC cases were caused by *C. albicans*. In summary, of the identified yeasts in the 15 patients, nine were *C*. *albicans*, three were *C*. *glabrata*, two were *C. dubliniensis*, and one was *C. tropicalis*. None of the patients had candidiasis in the Collection A or Collection B consecutive collection (Table 1). Notably, two patients had species substitutions between the Collection A and Collection B (Table 1).

**Table 1:** Oral condition, type of candidiasis, and *Candida* species identified in patients who underwent liver transplantation

Patients	Collection A		Collection B	
	Oral condition	Species	Oral condition	Species
P18	PC	*C. glabrata*	Col	*C. glabrata*
P26	AC	*C. albicans*	Col	*C. albicans*
P38	PC	*C. tropicalis*	Col	*C. glabrata*
P47	PC	*C. glabrata*	Col	*C. glabrata*
P87	PC	*C. glabrata*	Col	*C. albicans*
P33	PC	*C. albicans*	Col	*C. albicans*
P79	AC	*C. albicans*	Col	*C. dubliniensis*
P61	AC	*C. tropicalis*	Col	*C. tropicalis*
P72	Col	*C. albicans*	CA	*C. albicans*
P07	Col	*C. albicans*	PC	*C. albicans*
P20	Col	*C. dubliniensis*	CA	*C. dubliniensis*
P57	Col	*C. albicans*	CA	*C. albicans*
P85	Col	*C. albicans*	PC	*C. albicans*
P28	Col	*C. albicans*	CA	*C. albicans*
P43	Col	*C. albicans*	CA	*C. albicans*

P: patient; AC: atrophic candidiasis; PC: pseudomembranous candidiasis; Info: oral infection; Col: oral colonization.

Susceptibility tests were performed on 15 yeast isolates from Collections A and B, present in patients with candidiasis infection or colonization. All MIC variations, MIC_50_ and MIC_90_ values, and susceptibility categories are listed in table 2. The MIC_90_ values for *C. glabrata*, *C. tropicalis*, and *C. dubliniensis* were not determined because the number of isolates was deficient.

**Table 2:** Antifungal susceptibility profile of isolates of *Candida* spp. of patients with oral candidiasis who underwent liver transplantation

Species	Collection A (n=15)						
	Antifungal	MIC Variation (μg/mL)	MIC_50_ (μg/mL)	MIC_90_ (μg/mL)	Sensitive n (%)	SDD n (%)	Resistant n (%)
*C. albicans* (n=9)	Fluconazole	0.125-8	0.25	4	8 (88.9)	1 (11.1)	-
	Amphotericin B	0.25-2	0.5	1	9 (100)	-	-
	Micafungin	0.03-0.5	0.03	0.03	9 (100)	-	-
*C. glabrata* (n=3)	Fluconazole	0.125-4	0.25		-	3 (100)	-
	Amphotericin B	0.25-1	1		3 (100)	-	-
	Micafungin	0.03-0.5	0.03		3 (100)	-	-
*C. dubliniensis* (n=1)	Fluconazole	0.5	0.5		1 (100)	-	-
	Amphotericin B	0.5	0.5		1 (100)	-	-
	Micafungin	0.03	0.03		1 (100)	-	-
*C. tropicalis* (n=2)	Fluconazole	4-8	4		-	1 (50)	1 (50)
	Amphotericin B	0.5-1	0.5		2 (100)	-	-
	Micafungin	0.03-0.03	0.03		2 (100)	-	-
	**Collection B (n=15)**						
*C. albicans* (n=9)	Fluconazole	0.125-8	0.25	8	6 (66.7)	2 (22.2)	1 (711.1)
	Amphotericin B	0.25-2	0.5	1	9 (100)	-	-
	Micafungin	0.03-0.5	0.03	0.03	9 (100)	-	-
*C. glabrata* (n=3)	Fluconazole	0.5-1	0.5		-	3 (100)	-
	Amphotericin B	0.25-1	1		3 (100)	-	-
	Micafungin	0.03-0.5	0.03		3 (100)	-	-
*C. dubliniensis* (n=2)	Fluconazole	0.125-2	0.5		2 (100)	-	-
	Amphotericin B	0.5-1	0.5		2 (100)	-	-
	Micafungin	0.03-0.03	0.03		2 (100)	-	-
*C. tropicalis* (n=1)	Fluconazole	4	4		-	1 (100)	-
	Amphotericin B	0.06	0.06		1 (100)	-	-
	Micafungin	0.03	0.03		1 (100)	-	-

MIC: minimum inhibitory concentration; C: *Candida*; SDD: dose-dependent sensitivity; MIC_50_: antifungal concentration able to inhibit 50% of all isolates tested; MIC_90_: antifungal concentration able to inhibit 90% of all isolates tested.

All the tested isolates showed low MICs for amphotericin B and micafungin, suggesting that *Candida* spp. have in vitro susceptibility to these two antifungal agents. There are no defined breakpoints for *C. dubliniensis* concerning micafungin; therefore, we used the breakpoint values of *C. albicans* complex as a reference to analyze the results,^(20)^ thereby demonstrating that this species was susceptible to the drugs in both collections.

Fluconazole was the only antifungal agent with different susceptibility patterns in the studied yeasts. In Collection A, one isolate of *C. albicans*, three of *C. glabrata*, and one of *C. tropicalis* were susceptible to fluconazole in a dose-dependent manner, one *C. tropicalis* was resistant, and the others were susceptible to all antifungal agents.

In Collection B, two isolates of *C. albicans*, three of *C. glabrata*, and one of *C. tropicalis* were susceptible to fluconazole in a dose-dependent manner; one *C. albicans* isolate was resistant, and the others were susceptible to all antifungal agents. Although there are no defined breakpoints for *C. dubliniensis*, a rare and cryptic species, all MIC values suggest susceptibility.

All the patients with oral candidiasis were previously treated with topical nystatin for 30 days. One patient with CA presented with a fluconazole-resistant *C. tropicalis* isolate in the Collection A. Despite the nystatin treatment, in the Collection B, this patient presented with *C. glabrata* colonization after treatment with fluconazole (MIC = 8μg/mL).

In the Collection A, one patient presented with CA caused by *C. glabrata* and presented with a fluconazole-resistant isolate of *C. albicans* after six months. In another patient with PC, *C. tropicalis* isolates susceptible to fluconazole in a dose-dependent manner were identified in Collection A. After six months, the patient remained colonized by the same isolate of *C. tropicalis*.

## DISCUSSION

This descriptive study aimed to verify the presence of candidiasis in patients who underwent liver transplantation, identify the species causing the disease, and test the efficacy of each class of antifungal agents commonly used in therapy for invasive fungal infections caused by yeasts. We analyzed 97 patients six months post-liver transplantation, and 15.47% of them presented with oral candidiasis during two collection periods within a six-month interval, showing that this is an opportunistic infection that can occur at any time post-transplantation. This finding aligns with a similar study conducted in Iran, which identified candidiasis in 16% of patients who underwent liver transplantation.^(21)^ Furthermore, previous studies’ results demonstrated that the prevalence of oral candidiasis in patients who have undergone solid organ transplants ranges from 15-34%.^(22-25)^ This study found a higher prevalence of candidiasis than López-Pintor et al.,^(26)^ who reported candidiasis infection in 7.4% of patients who underwent kidney transplantation, indicating that the incidence of fungal infections is higher in patients receiving liver transplantation. Notably, the frequency of oral and oropharyngeal candidiasis infections has increased dramatically in recent decades, mainly because of an increase in the number of patients with compromised immune systems.^(25)^

In the present study, most patients (53.4%) had AC, similar to that observed in other studies. Zhang et al.^(27)^ showed that patients with complete dentures who underwent kidney transplantation were more prone to candidiasis and angular cheilitis than healthy individuals with the same type of prosthesis. *Candida albicans* is responsible for most cases of oral candidiasis infections; however, other species, such as *C. glabrata, C. krusei, C. tropicalis, C*. *parapsilosis*, and *C. dubliniensis*, have been frequently identified as causing the disease.^(28)^ In this study, of the 15 cases of candidiasis, *C. albicans* (63.9%) was the most prevalent species causing infections, followed by *C. glabrata* (18.5%). Similar data were found in previous studies in various regions worldwide.^(3,25,27)^

*Candida glabrata* and *C. tropicalis* are the second and third most prevalent species identified in this study (25.3%), similar to studies conducted by Sahebjamee et al.^(21)^ and Zarei et al.,^(25)^ which found that approximately 22% of *candidiasis* infections not caused by *C. albicans* are caused by these two species. The present study identified one case of candidiasis caused by *C. dubliniensis*, which is consistent with the findings of Chavasco et al.,^(29)^ who also identified this species in two cases of oral candidiasis.^(29)^

A study of HIV-positive children in Chile showed that *C. glabrata* (33%), followed by *C. albicans* (27%), was the most common cause of oral candidiasis infection. Castillo-Martínez et al.^(30)^ identified oral candidiasis in 14% of patients who underwent bone marrow transplantation, and Nishii et al.^(31)^ identified oral candidiasis in 41.7% of patients who underwent radiotherapy in a study in Japan.

The number of *C. albicans* isolates decreased, and the number of *C*. *glabrata* and *C. parapsilosis* isolates increased over time in cases of invasive candidiasis.^(14,32)^ The most prevalent species in the present study and most other studies was *C*. *albicans,* with it being responsible for approximately 50% of all infections; however, *C. glabrata*, *C. tropicalis*, *C. parapsilosis*, and *C. krusei* are also important infectious agents.^(6,33-39)^

The present study identified *C. dubliniensis* in 8.5% of patients using a species-specific PCR to differentiate this species from *C. albicans*. Chavasco et al.^(29)^ identified *C. dubliniensis* in 5.4% of patients using PCR, in which samples had been mistakenly identified as *C. albicans* by the classical method, thus demonstrating that PCR is more effective in elucidating the epidemiology of *C. dubliniensis* and establishing its clinical significance.

Recently, antifungal susceptibility testing has revealed new agents and new standardized test methods have been developed by the Antifungal Susceptibility Testing Subcommittee of the CLSI^(18)^ and European Committee of Antimicrobial Susceptibility Testing (EUCAST),^(19)^ through which the knowledge of susceptibility can be improved, especially for opportunistic pathogens.^(14)^

In the present study, one colonizing isolate of *C. albicans* and *C. tropicalis* that caused infection showed resistance to fluconazole. Goular et al.^(28)^ found fluconazole-resistant *Candida* species in 1% of all strains observed, which is consistent with the findings of this study. Orasch et al.^(38)^ found fluconazole susceptibility in 90% of the strains of *C. albicans, C. glabrata, C. tropicalis*, and *C. parapsilosis*; Pfaller et al.^(14)^ observed the highest rates of fluconazole-resistant isolates in *C. glabrata* in North America (10.6%) and in *C. tropicalis* in the Asia-Pacific region (9.2%), and reported a steady increase in fluconazole-resistant isolates of *C. glabrata* throughout 20 years of studies in the United States. For echinocandins, resistance results ranged from 3.5% for *C. glabrata* to 0.1% for *C. albicans* and *C. parapsilosis*, which were lower than those in this study. A study by Orasch et al.^(38)^ reported high micafungin resistance in *C. glabrata* (2.8%) and *C. tropicalis* (1.3%). Modrzewska et al.^(39)^ revealed that almost all strains were susceptible to nystatin (97.9%) and amphotericin B (72.3%) and resistant to fluconazole (72.3%) and ketoconazole (57.5%). Therefore, it is necessary to determine the fungal species and their antifungal susceptibility for more effective therapy. Prophylactic administration of fluconazole often leads to an increase in the number of fluconazole-resistant strains.^(39)^ In this study, most isolates were susceptible to all antifungal agents; however, one isolate of *C. tropicalis* and *C. albicans* was resistant to fluconazole, suggesting a risk of invasive fungal infection by yeasts resistant to this drug, which were present in the oral cavity of patients.

Despite our promising results, this study had two limitations. First, it was not possible to standardize the time of the first post-transplant collection for all patients owing to the study design, and it was only possible to standardize the six-month interval between the two collection periods. Second, there was a lack of patient history and clinical information regarding the previous use of azoles. However, we believe this study makes a significant contribution to the existing literature, as it is the first regional investigation to determine the prevalence of oral candidiasis and accurately identify the species causing infections based on molecular methods, as well as their behavior against the action of azoles. In addition, this study offers insights for clinical staff on the emergence of in vitro-antifungal-resistant strains, which may enable more effective treatment of patients with oral candidiasis. Furthermore, this study reports the emergence of resistance in these species, which may result from increased use of azoles in prophylactic therapy.

## CONCLUSION

The primary type of candidiasis identified in patients who underwent liver transplantation is atrophic candidiasis, with the most prevalent causative species being *C. albicans* and *C. glabrata*. Even in the absence of clinical signs of candidiasis, all patients had an oral cavity colonized by species of *Candida*, either before or after infection and treatment, demonstrating that this yeast was common in the patients who participated in this study. Most isolates were susceptible to antifungal agents; however, one isolate of *C. tropicalis* and *C. albicans* was resistant to fluconazole.

## References

[1] Ahmed R, Sharma A, Halawa A (2019). Immunosuppression and Oral Health; the Implications of Organ Transplantation. JRTS.

[2] Pappas PG, Kauffman CA, Andes DR, Clancy CJ, Marr KA, Ostrosky-Zeichner L (2016). Clinical practice guideline for the management of CAndidiasis: 2016 update by the Infectious Diseases Society of America. Clin Infect Dis.

[3] Simms-Waldripa T, Rosenb G, Nielsen-Sainesc K, Ikedaa A (2015). Browna & Theodore Moorea. Invasive Fungal Infections in Pediatric Solid Organ Transplant Patients: epidemiology and Management. Curr Fungal Infect Rep.

[4] Badiee P, Jafarian H, Malek-Hosseini SA, Ghasemmi F (2019). Severe Cutaneous Candidiasis in a Liver Transplant Patient. Int J Organ Transplant Med.

[5] Sganga G, Bianco G, Frongillo F, Lirosi MC, Nure E, Agnes S (2014). Fungal infections after liver transplantation: incidence and outcome. Transplant Proc.

[6] Barahona-Correa JE, Calvo-Valderrama MG, Romero-Alvernia DM, Angulo-Mora J, Alarcón-Figueroa LF, Rodríguez-Malagón MN (2019). Epidemiology of Candidemia at a University Hospital in Colombia, 2008-2014. Univ Med.

[7] Quindós G (2014). Epidemiology of candidaemia and invasive candidiasis. A changing face. Rev Iberoam Micol.

[8] Botero MC, Puentes-Herrera M, Cortés JA (2014). Lipid formulations of amphotericin. Rev Chilena Infectol.

[9] Kaufman D, Liken H, Odackal N (2019). Diagnosis, Risk Factors, Outcomes, and Evaluation of Invasive Candida Infections. Infect Dis.

[10] Treister N, Duncan C, Cutler C, Lehmann L (2012). How we treat oral chronic graft-versus-host disease. Blood.

[11] López-Martínez R (2010). Candidosis, a new challenge. Clin Dermatol.

[12] Sanguinetti M, Posteraro B, Lass-Flörl C (2015). Antifungal drug resistance among Candida species: mechanisms and clinical impact. Mycoses.

[13] Cleveland AA, Farley MM, Harrison LH, Stein B, Hollick R, Lockhart SR (2012). Changes in incidence and antifungal drug resistance in candidemia: results from population-based laboratory surveillance in Atlanta and Baltimore, 2008-2011. Clin Infect Dis.

[14] Pfaller MA, Diekema DJ, Turnidge JD, Castanheira M, Jones RN (2019). Twenty Years of the SENTRY Antifungal Surveillance Program: Results for Candida Species From 1997-2016. Open Forum Infect Dis.

[15] Sabadin CE, Lopes SL, Gompertz OF, Santana GN, Azevedo Melo AS, Rigo L (2020). Oral colonization by Candida spp. in liver transplant patients: Molecular identification and antifungal susceptibilityOral colonization by Candida spp. in liver transplant. Med Mycol.

[16] Merseguel KB, Nishikaku AS, Rodrigues AM, Padovan AC, Ferreira RC, Azevedo Melo AS (2015). Genetic diversity of medically important and emerging Candida species causing invasive infection. BMC Infect Dis.

[17] Ahmad S, Khan Z, Asadzadeh M, Theyyathel A, Chandy R (2012). Performance comparison of phenotypic and molecular methods for detection and differentiation of Candida albicans and Candida dubliniensis. BMC Infect Dis.

[18] Clinical and Laboratory Standards Institute (CLSI) (2017). Reference method for broth dilution antifungal susceptibility testing of yeasts. CLSI standard M27.

[19] Clinical and Laboratory Standards Institute (CLSI) (2017). Performance standards for antifungal susceptibility testing of yeasts. CLSI supplement M60.

[20] Nguyen MH, Clancy CJ, Yu VL, Yu YC, Morris AJ, Snydman DR (1998). Do in vitro susceptibility data predict the microbiologic response to amphotericin B? Results of a prospective study of patients with Candida fungemia. J Infect Dis.

[21] Sahebjamee M, Shakur Shahabi M, Nikoobakht MR, Momen Beitollahi J, Mansourian A (2010). Oral lesions in kidney transplant patients. Iran J Kidney Dis.

[22] Olczak-Kowalczyk D, Pawlowska J, Garczewska B, Smirska E, Grenda R, Syczewska M (2010). Oral candidiasis in immunosuppressed children and young adults after liver or kidney transplantation. Pediatr Dent.

[23] Rodríguez ML, Rosa CA, Rodríguez JG, Nastri ML, Jewtuchowicz VM (2018). The Oral Cavity: A Reservoir that Favors Colonization and Selection of Candida parapsilosis sensu stricto Strains with High Pathogen Potential Under Conditions of Gingival-periodontal Disease. J Dent Sci Ther.

[24] Nishii M, Soutome S, Kawakita A, Yutori H, Iwata E, Akashi M (2020). Factors associated with severe oral mucositis and candidiasis in patients undergoing radiotherapy for oral and oropharyngeal carcinomas: a retrospective multicenter study of 326 patients. Support Care Cancer.

[25] Zarei F, Hashemi SJ, Salehi M, Mahmoudi S, Zibafar E, Ahmadinejad Z (2020). Molecular characterization of fungi causing colonization and infection in organ transplant recipients: a one-year prospective study. Curr Med Mycol.

[26] López-Pintor RM, Hernández G, de Arriba L, de Andrés A (2013). Oral candidiasis in patients with renal transplants. Med Oral Patol Oral Cir Bucal.

[27] Zhang XB, Yu SJ, Yu JX, Gong YL, Feng W, Sun FJ (2012). Retrospective analysis of epidemiology and prognostic factors for candidemia at a hospital in China, 2000-2009. Jpn J Infect Dis.

[28] Goulart LS, Souza WW, Vieira CA, Lima JS, Olinda RA, Araújo C (2018). Colonização oral por espécies de Candida em pacientes HIV positivo: estudo de associação e suscetibilidade antifúngica. einstein (Sao Paulo).

[29] Chavasco JK, Paula CR, Hirata MH, Aleva NA, Melo CE, Gambale W (2006). Molecular identification of Candida dubliniensis isolated from oral lesions of hiv-positive and hiv-negative patients in São Paulo, Braazil. Rev Inst Med Trop São Paulo.

[30] Castillo-Martínez NA, Mouriño-Pérez RR, Cornejo-Bravo JM, Gaitán-Cepeda LA (2018). Factores relacionados a candidiasis oral en niños y adolescentes con VIH, caracterización de especies y susceptibilidad antifúngica. Rev Chilena Infectol.

[31] Nishii M, Soutome S, Kawakita A, Yutori H, Iwata E, Akashi M (2020). Factors associated with severe oral mucositis and candidiasis in patients undergoing radiotherapy for oral and oropharyngeal carcinomas: a retrospective multicenter study of 326 patients. Support Care Cancer.

[32] da Matta DA, Souza AC, Colombo AL (2017). Revisiting Species Distribution and Antifungal Susceptibility of Candida Bloodstream Isolates from Latin American Medical Centers. J Fungi (Basel).

[33] Kubak BM, Huprikar SS, AST Infectious Diseases Community of Practice (2009). Emerging & rare fungal infections in solid organ transplant recipients. Am J Transplant.

[34] Falagas ME, Roussos N, Vardakas KZ (2010). Relative frequency of albicans and the various non-albicans Candida spp among candidemia isolates from inpatients in various parts of the world: a systematic review. Int J Infect Dis.

[35] Fabuel LC, Esteve CG, Pérez MG (2011). Dental management in transplant patients. J Clin Exp Dent.

[36] Silva-Rocha WP, Lemos VL, Svidizisnki TI, Milan EP, Chaves GM (2014). Candida species distribution, genotyping and virulence factors of Candida albicans isolated from the oral cavity of kidney transplant recipients of two geographic regions of Brazil. BMC Oral Health.

[37] Sganga G, Bianco G, Frongillo F, Lirosi MC, Nure E, Agnes S (2014). Fungal infections after liver transplantation: incidence and outcome. Transplant Proc.

[38] Orasch C, Marchetti O, Garbino J, Schrenzel J, Zimmerli S, Mühlethaler K, Pfyffer G, Ruef C, Fehr J, Zbinden R, Calandra T, Bille J, FUNGINOS (2014). Candida species distribution and antifungal susceptibility testing according to European Committee on Antimicrobial Susceptibility Testing and new vs. old Clinical and Laboratory Standards Institute clinical breakpoints: a 6-year prospective candidaemia survey from the fungal infection network of Switzerland. Clin Microbiol Infect.

[39] Modrzewska BD, Kurnatowska AJ, Khalid K (2017). Drug susceptibility of fungi isolated from ICU patients. Ann Parasitol.

